# Partner familiarity enhances performance in a manual precision task

**DOI:** 10.1038/s41598-025-03341-9

**Published:** 2025-07-02

**Authors:** Johannes Heidersberger, Jakob Kaiser, Shail Jadav, Lucija Mihić Zidar, Arianna Curioni, Leif Johannsen, Dongheui Lee

**Affiliations:** 1 Autonomous Systems Lab, Institute of Computer Technology, TU Wien, Vienna, Austria; 2 Nuremberg Institute for Market Decisions, Nuremberg, Germany; 3 Faculty of Psychology, University of Vienna, Vienna, Austria; 4 Department of Cognition and Experimental Psychology, Institute of Psychology, RWTH Aachen University, Aachen, Germany; 5 Institute of Robotics and Mechatronics, German Aerospace Center (DLR), Weßling, Germany

**Keywords:** Partner familiarity, Collaborative learning, Joint action, Human-human interaction, Cooperation, Motor control, Sensorimotor processing

## Abstract

Understanding human collaborative behavior in tasks with physical interaction is essential for advancing physical human-robot collaboration. Investigating how individuals learn to collaborate over repeated interactions can provide valuable insights for developing robotic agents capable of gradually improving coordination and collaboration performance. Therefore, this study investigated learning behavior in a high-precision task over repeated haptic collaboration. Specifically, we examined if learned collaboration behavior is partner-specific, what collaboration strategies are developed, and if interpersonal differences affect collaboration. Our results indicate that repeated physical collaboration with the same partner allowed for immediate high performance with a familiar partner in subsequent collaborations, whereas adapting to an unfamiliar partner required retraining. Participants used partner-specific collaboration behaviors—in terms of motions and forces—that could be retained in subsequent interactions. Collaborators reduced the variability of their arm motions over repeated collaboration, achieving higher performance, likely due to increased predictability. Collaboration also enabled knowledge transfer between partners, with individual improvement being enhanced when paired with a better-performing partner. These findings suggest that partners in a collaborative precision task optimize their performance by gradually negotiating a joint action strategy, which is reused in subsequent collaborations with familiar partners and carries over to solo task execution.

## Introduction

Collaborative haptic interactions are essential for performing various tasks, from moving heavy objects to performing precision surgeries. In precision physical collaboration tasks, such as surgery, successful collaboration requires that the actions of haptically interacting partners are precisely coordinated. Haptic communication—information exchange via forces and movements—has been shown to be essential for such interpersonal coordination during physical interaction^1,2^. The study of physical human-human collaboration has attracted increasing attention due to its potential applications in physical human-robot collaboration (pHRC)^3,4^. In pHRC, advantages of both the human and the robot can be leveraged to achieve increased task performance^4,5^. Understanding how humans learn to collaborate over repeated interactions may enable the development of robots that gradually learn to complement the partner’s behavior to improve coordination and, consequently, enhance collaboration performance. The current study investigates how physically collaborating individuals learn to collaborate in a repeated high-precision task.

Learning a forward model of a partner’s collaborative behavior over repeated interactions would allow individuals to predict their partner’s actions^6,7^, potentially improving the predictability of joint actions and enhancing coordination. Such a predictive model would support two key cognitive processes: on the one hand, self-other distinction, which enables the differentiation between self-generated and partner-caused effects; on the other hand, self-other integration, which merges both partners’ actions into a coordinated, shared goal^8^. By integrating the predictions of the partner’s actions into their own action planning, collaborators can coordinate their individual actions^9^. This emphasizes the importance of considering the partner’s intention, e.g., in the form of a movement goal, when planning and adjusting one’s own behavior is one aspect of successful collaboration^10^. Additionally, based on predictions of a partner’s behavior, collaborators can also distinguish who is responsible for certain parts of the task and when it is appropriate to act^11^, enabling a separation into complementary behaviors to achieve a common goal^3^. Learning a predictive model of the particular partner’s contribution would imply limited transferability to unfamiliar partners. Adaptation behavior specific to the partner has been observed in social interactions, such as during dialog^12^. It remains to be investigated if collaboration knowledge learned in physical collaboration tasks is specific to the partner with whom it is learned and, therefore, if the transferability of this knowledge to an unknown partner is limited^13^. We, therefore, investigate if familiarity with a partner improves physical collaboration (H1) and if individuals acquire partner-specific interaction dynamics during repeated physical collaboration (H3).

In collaboration tasks, reducing action variability has been identified as a common strategy to enhance predictability and, consequently, collaboration performance^14–16^. Human collaborators in non-physical tasks lower the temporal variability in their actions, leading to higher predictability of the timing of their actions^14,17^. In physical interaction tasks, reducing variability in both the timing and magnitude of applied forces has been shown to improve collaboration performance^15,18^. Additionally, by maintaining a consistent action sequence, the predictability and, consequently, the performance can be increased^16^. However, to our knowledge, the collaboration strategy of increasing predictability through action variability reduction has not yet been examined in physical tasks with continuously high precision requirements. Thus, we examine if collaborators learn to reduce action variability over consecutive trials of a high-precision task to achieve higher collaboration performance (H2).

Research in task learning has shown that learned knowledge can be retained over extended periods, allowing individuals to quickly achieve previous performance levels when re-engaging with the task^19,20^. However, task retention is influenced by task demands, with explicit information and physical tasks being retained better than sensorimotor or accuracy-focused aspects^20,21^. Physical human-human collaboration can improve the retention of task performance over training the task alone^22^. However, current robotic collaboration agents are unable to improve skill retention beyond the level of training the task alone^22^. This shows that the mechanisms behind high skill retention in human-human collaboration are not fully understood, although understanding them is essential for the development of robotic agents with comparable retention benefits. Moreover, if certain aspects of physical collaboration behavior are specific to the partner with whom one is collaborating, the question arises whether these partner-specific behaviors can be retained over time. We investigate if learned interaction dynamics that may be specific to the collaboration partner can be retained (H3).

Understanding how collaboration affects performance and facilitates knowledge transfer between individuals is a key aspect of research on human collaboration. Previous studies have indicated that by sharing control over degrees of freedom, collaborators can exceed solo performance levels, while similar benefits cannot be achieved if control is distributed between partners^1,13,23^. Studies examining the effect of performance differences between collaborators report varying outcomes: larger individual performance differences were found to hinder collaborative performance^24^, whereas partnering with an expert improved collaborative performance in other cases^1,25^. During collaboration, the benefits for the lower-performing partner may come at the cost of the higher-performing partner^26^. Research shows inconclusive results on collaboration’s effect on individual performance, with one study reporting improvement in individual performance^1^ and others finding no effect^23,27^. Compared to collaboration with an expert, partnering with a collaborator of a similar performance level has been shown to enhance individual learning^25,28^. These findings emphasize the need for further research to clarify how task-specific factors and partner characteristics influence collaborative learning and performance outcomes. We study whether interpersonal differences, such as individual performance levels and anthropometrics, affect collaboration performance and learning capabilities (H4).

In this work, we investigate learning behavior over repeated physical collaboration using a collaborative hot wire task. In this task, pairs of participants had to move a manipulandum along a predefined wire path without making contact with the wire, demanding precise control of the object’s motion. Effective collaboration in the hot wire task requires partners to coordinate their shared control over the object’s motion precisely in space and time, enabling them to jointly execute precise corrective actions to rectify position errors. For every movement adjustment when performing the task, participants will experience a distinction between self-induced action effects and the action effects caused by the interaction partner. This is perceived haptically through interaction forces and object movement. However, due to the high precision demands the capability to adapt motions is limited, which constrains haptic communication used for interpersonal coordination during physical interaction^1,2^. Any perceived effects that cannot be explained or do not match with expectations of own action effects may then be attributed to the interaction partner. Gradually, a predictive model of the partner’s collaboration behavior may be acquired through repeated exposure to the contribution of an interaction partner during the joint action^6,7^. The knowledge acquired about the interaction partner may enhance spatiotemporal movement coordination between both interaction partners^29^ and could facilitate self-other integration, which may lead to increased joint action predictability. Additionally, this acquired knowledge may facilitate self-other distinction, which could help in learning to perform actions that complement the partner’s actions.

In summary, we investigate (H1) whether partner familiarity improves collaboration performance, (H2) whether action variability gets reduced to improve collaborative performance, (H3) whether partner-specific interaction dynamics are learned and retained, and (H4) how interpersonal differences influence collaboration performance and learning capabilities. By focusing on these aspects, this study contributes to a deeper understanding of the learning behavior within physical collaboration, which may offer insights that could inform the development of more effective human-robot interactions in tasks requiring high-precision haptic coordination.

## Methods

### Participant information

In this study, we tested 44 participants. These participants had a mean age of 25.57 years (standard deviation $SD = 4.51$ years). The gender distribution was 30 females, 13 males, and one participant identifying as other. Regarding handedness, 36 participants were right-handed, seven were left-handed, and one was ambidextrous. The mean height of participants was 169.86 cm ($SD = {9.42}\,{\hbox {cm}}$), and the mean weight was 65.89 kg ($SD = {13.49}\,{\hbox {kg}}$). None of the participants had prior experience with the task before taking part in the experiment. A power analysis based on^30^ was performed for the statistical model with Eq. (6), which investigates the effects of trial progression and collaboration conditions on the task performance of collaborating participants. The analysis showed that with effect sizes of 0.5 for trials and 15.0 for conditions, this sample size was sufficient to achieve a power greater than 0.85 at a significance level of 0.05. In the study, 44 unique participant pairs were formed throughout the experiment (see section “Experiment structure” for details on how pairs were arranged). Among them, 30 pairs consisted of two right-handed participants, two pairs included two left-handed participants, 10 pairs combined one left- and one right-handed individual, and two pairs consisted of one right-handed and one ambidextrous participant. Regarding gender composition, there were 6 male-male pairs, 14 male-female pairs, 22 female-female pairs, and 2 pairs involving a participant identifying as other (paired once with a female and once with a male). Participants were informed about the purpose of the study, the procedure, potential risks, and their rights regarding participation and data protection. All participants confirmed their participation in the study by signing a written informed consent form. The study was performed in accordance with relevant guidelines and regulations. The ethics proposal for this study was approved by the Pilot Research Ethic Committee (Pilot REC) of TU Wien (case number: 024_21102022).

**Fig. 1:**
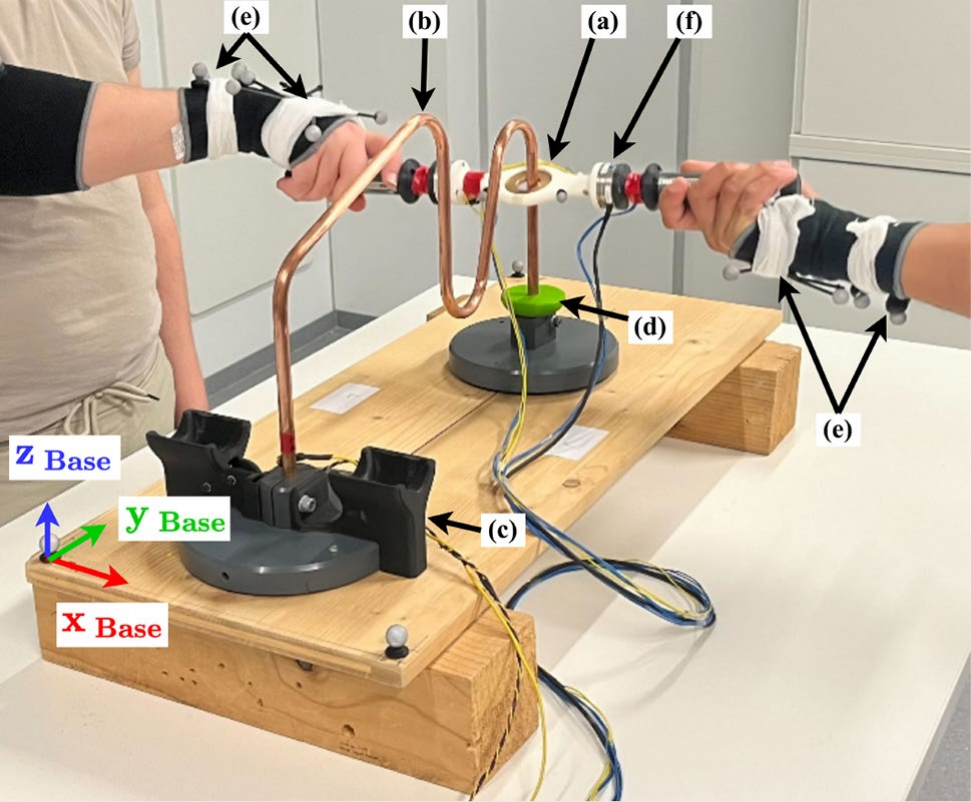
Experimental setup of the hot wire apparatus used in this study. One trial consists of the collaborators moving the handle (**a**) along the hot wire path (**b**) from the docking station (**c**) to the turning point (**d**) and back. The objective is to finish the trail within a specified amount of time while minimizing the percentage of time the handle is in contact with the wire. Motion tracking markers were placed on the handle (**a**) and participants’ hands and wrists (**e**). A force-torque sensor (**f**) was integrated into the handle for measuring interaction forces.

### Experiment structure

The experiment was structured into five blocks as described in Table 1 and was conducted in groups of four participants (referred to as Participants P1 to P4 in the following). Each participant first performed the task alone one after another (Solo condition S1) to establish a baseline for individual performance prior to collaboration. Then, they executed the task in fixed pairs to train the collaboration (Participant P1 with Participant P2, Participant P3 with Participant P4) during the first joint block (Training condition A1). This was followed by a second solo block (S2), with the same order of participants as in S1, to investigate any transfer of performance between individuals due to the preceding collaboration. The final two blocks were joint tasks performed either with the same partner as in condition A1 (Repeat condition A2) or with an unknown partner (Switch condition B, where Participant P1 was paired with Participant P3 and Participant P2 with Participant P4). In condition B, both participants had prior collaboration experience, but with a different partner, requiring both to adapt to their new interaction partner. The order of A2 and B was randomized across groups to avoid the influence of order effects when comparing these conditions. Due to this structure, all participants had the same amount of prior task exposure when collaborating. When transitioning from one block condition to the other, participants had a break, except for the transition from the second to the third joint block. Here, the duration of the intermediate breaks varied depending on the participant due to the changing pairings. These differences balance each other out and should therefore not affect our results when comparing the block conditions. This design resulted in 44 unique pairs for the experiment, with 24 participants completing block order 1 and 20 participants completing block order 2. Each solo block consisted of five trials, while the joint blocks comprised fifteen trials. The average durations of the blocks were as follows: S1 – 10.11 min, A1 – 11.13 min, S2 –8.04 min, A2 – 10.16 min, and B – 10.14 min.

**Table 1:**
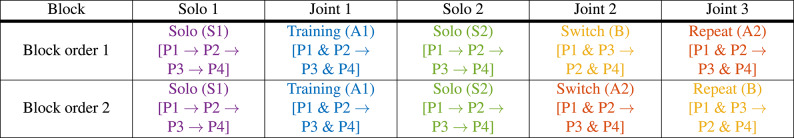
Structure of the experiment with two different block orders consisting of solo (S1 and S2) and collaboration blocks (A1, A2, and B).

The entries in square brackets indicate the order in which participants (P1-P4) performed each block, with arrows showing the sequence of individual or paired participation.

### Experiment procedure

As a collaborative task with high precision requirements and interpersonal haptic interaction, a collaborative version of the hot wire game was chosen, where a metal ring must be moved along a wire path while trying to avoid contact between the ring and the wire (see Fig. 1). Participant data, i.e., age, height, weight, and gender, were collected using an anonymized form at the start of each experiment session. Before the start of the experiment, participants were instructed to hold the handle with their right hand in a specific way that allowed them to perform the full movement without requiring them to change the grip during trial execution. As shown in Fig. 1, the participant starting the motion from the left gripped the handle from the top, while the other participant gripped it from the bottom, enabling to perform the required handle rotation to reach the contact point. The participants were further instructed to stand directly opposite each other (face-to-face configuration), facing the wire path between them, allowing them to perform the full range of motion required for the task without stepping laterally. They were asked to maintain their standing position and not to lean on the table. During the solo trials in conditions S1 and S2, participants controlled the handle by holding only the grip located on their side, while the grip on the opposite side remained unused. Detailed information about the experiment structure, including the different block conditions and the number of trials, was provided to participants as well. Participants were informed that they had a target time of 20 s to complete each trial and should aim for a steady and comfortable pace. At the beginning of each trial, a high-pitched sound was played after which the participants holding the handle were allowed to start the trial by lifting the handle from the docking station. In each trial, participants had to move the handle to the hot wire’s turning point, touch a contact piece, and then return it to the start position, while trying to avoid contact with the wire throughout the whole motion. To end the trial, the handle had to be placed in the docking station, whereafter a low-pitched sound was played to signal the trial stop to the participant. After each trial, a score value ranging from 0 to 100 was displayed, reflecting the task performance (see Eqs. (1–3) for details on the calculation of the score value). Participants were asked to review their score in order to improve the task performance in subsequent trials. After each experimental block, the participants filled out a questionnaire that included all six items from the NASA TLX assessment tool^31^—mental demand, physical demand, temporal demand, performance, effort, and frustration—as well as additional questions on the sense of control and perceived task contribution. For the present analysis, we examined the questions regarding temporal task load, sense of control, subjective performance, and perceived contribution relative to their partner’s contribution (see Table 2). The contribution question was only asked after collaboration blocks (A1, A2, and B). The average duration of the experiment was 1 hour and 33 min ($SD = 5$ min), including calibration, execution of the trials, and filling out the questionnaires. The mean trial duration was 23.05 s ($SD = 7.75$ s).

**Table 2:** Questions from the questionnaire that participants were asked after each experimental block.

Question	Abbreviated description	Scale
How hurried or rushed was the pace of the task?	Temporal load	− 4 (Low) to 4 (High)
How successful were you in accomplishing what you were asked to do?	Performance	− 4 (Low) to 4 (High)
How much did you feel in control during the task?	Sense of control	− 4 (Low) to 4 (High)
Who contributed more to the task?	Contribution	− 4 (My Partner) to 4 (Me)

### Experiment measurements

For each trial, we measured the trial execution time, the contact state between the handle and the wire, the interaction forces and torques, and the movements of the handle and the human hands and wrists. Additionally, gripping strength and movements of the elbow and shoulder were recorded, although these were not included in the subsequent analysis. These measures were recorded at 20 Hz. In our specific setup, a copper pipe with an outer diameter of 10 mm was bent to a shape of 1 m length as depicted in Fig. 1. The handle, weighing 368 g, consisted of a centerpiece and grips on opposing sides. In the centerpiece, a brass ring was recessed with a hole diameter of 20 mm, resulting in a 10 mm clearance between the ring and the wire. When the ring contacts the copper wire during task execution, an electrical circuit is closed, which was used to register the contact. A force-torque sensor (Schunk FTN-Mini-40 SI-80-4) was mounted between the centerpiece and one of the grips (Fig. 1(f)), measuring the forces and torques applied to the handle. To balance the weight distribution of the handle such that the center of mass was located in the center of the contact ring, a counterweight for the force-torque sensor was mounted on the opposing handle side. The handle’s motion began and ended in a docking station equipped with a switch that was activated by the handle’s weight. The trial’s execution time was measured from the moment the handle was lifted from the docking station until it was returned. A motion capture system from OptiTrack using twelve Prime^X^13 cameras was used to capture the motion of the handle and the arm motions of participants using rigid body trackers on the handle, hands, and wrists (see Fig. 1(a) and (e)). The trackers are placed on the participants’ right arms, as only the right hand is used to execute the task. All motions were represented in the base coordinate system, see Fig. 1.

### Metrics for data analysis

We considered metrics for task performance and time series similarity to evaluate the collected data.

#### Task performance

The score value $s \in \left[ 0, 100 \right]$, displayed to the participants after every trial, is used as a performance metric. Since a speed-accuracy tradeoff was evident from pilot data for this task, penalties for long execution times (time penalty) and imprecise movements indicated by contact between the handle and the hotwire (contact penalty) were deducted from the maximum score of 100.

1 $$\begin{aligned} s = {\left\{ \begin{array}{ll} 0 & \text {if } p_{\text {time}} + p_{\text {contact}} > 100 \\ 100 - p_{\text {time}} - p_{\text {contact}} & \text {if } p_\textrm{time} + p_\textrm{contact} \le 100 \end{array}\right. } \end{aligned}$$

The time penalty $p_\textrm{time} \in \left[ 0, 100 \right]$, which encourages the participants to finish within a given time, is calculated by multiplying the task execution time $t_\textrm{exec}$ exceeding a threshold of $t_\textrm{th} = {20}\,{\hbox {s}}$ by a constant scaling factor:

2 $$\begin{aligned} p_{\text {time}} = {\left\{ \begin{array}{ll} 0 & \text {if } t_{\text {exec}} \le t_{\text {th}} \\ {2} \frac{1}{{\hbox {s}}} (t_{\text {exec}} - t_{\text {th}}) & \text {if } t_{\text {th}}< t_{\text {exec}} < t_{\text {th}} + \frac{100}{{2}} {{\hbox {s}}} \\ 100 & \text {if } t_{\text {th}} + \frac{100}{{2}} {{\hbox {s}}} \le t_{\text {exec}} \end{array}\right. } \end{aligned}$$

The precision penalty $p_\textrm{contact} \in \left[ 0, 100 \right]$ is based on the percentage of time the handle’s ring is in contact with the wire $t_{\text {contact}}$ relative to the total execution time. This percentage value is then scaled by a constant factor to compute the penalty:

3 $$\begin{aligned} p_{\text {contact}} = {\left\{ \begin{array}{ll} {300} \frac{t_{\text {contact}}}{t_{\text {exec}}} & \text {if } \frac{t_{\text {contact}}}{t_{\text {exec}}} < \frac{100}{{300}} \\ 100 & \text {if } \frac{100}{{300}} \le \frac{t_{\text {contact}}}{t_{\text {exec}}} \end{array}\right. } \end{aligned}$$

The time threshold $t_\textrm{th}$ was determined based on the preceding piloting of the experiment task to select an achievable target time for completing the task. The scaling factors for the penalties were selected and validated during pilot experiments to reflect the task’s main objective of high movement precision by assigning higher penalties to contacts, while also ensuring appropriate task difficulty.

#### Time series similarity

We used a metric based on Dynamic Time Warping (DTW)^32^ to quantify how similar two compared time series are. When, e.g., comparing two motions that might follow the same path but with varying speeds, DTW can be used to remove these non-linearly varying speed differences. Given two time series $\mathbf{X}_a \in \mathbb {R}^{N_a \times M}$ and $\mathbf{X}_b \in \mathbb {R}^{N_b \times M}$, with sample numbers $N_a$ and $N_b$ and dimensionality *M*, the DTW algorithm finds the warping path $\pi _\textrm{DTW}$ which aligns the sequences such as to minimize a distance metric (here Euclidean distance) between both:

4 $$\begin{aligned} \pi _\textrm{DTW}(\mathbf{X}_a,\mathbf{X}_b) = \min _\pi \sum _{(i,j) \in \pi } \sqrt{\sum _{m=1}^{M} (X_{a,i,m} - X_{b,j,m})^2} \end{aligned}$$

This warping path consists of a set of index pairs $\left\{ \left( i_k , j_k \right) \right\} _{k=1}^{K}$ representing the alignment between samples from $\mathbf{X}_a$ and $\mathbf{X}_b$. Based on the warping path $\pi _\textrm{DTW}$ from Eq. (4) the dissimilarity between the aligned time series can be quantified with the DTW distance, computed as the sum of the Euclidean distances between the aligned samples. To additionally reduce the influence of varying time series lengths the DTW distance is normalized by the warping path length. This metric *d*, therefore, represents the average Euclidean distance of all aligned samples.

5 $$\begin{aligned} d(\mathbf{X}_a,\mathbf{X}_b)&= \frac{1}{K} \sum _{(i,j) \in \pi _\textrm{DTW}} \sqrt{\sum _{m=1}^{M} (X_{a,i,m} - X_{b,j,m})^2} \end{aligned}$$

## Results

First, we analyzed how familiarity with the partner affects the quantitative performance score and the subjective assessment of the collaboration. Next, we examined if collaborators increase the predictability of their actions to enhance the collaboration performance. We also investigated whether the interaction dynamics used during collaboration were specific to the partner. Finally, the effects of interpersonal differences on performance and learning capabilities were examined. We use Linear Mixed Effects Models (LMEMs)^33^ for the statistical analysis as they allow us to account for the non-independence of observations as we have in our experimental data due to the grouping of participants and conditions. Results are considered significant at a p-value of $p<0.05.$

### (H1) Partner-specific learning in a physical collaboration task

To analyze the effect of partner familiarity on collaboration, we first examine its impact on performance scores and joint improvement. For this analysis, we use an LMEM that captures the fixed effects of trial number and block condition while accounting for the random effect of a performance level specific to each pair:

6 $$\begin{aligned} \text {Score}_{klm} =&\beta _{\text {A1}} \text {Condition}_{\text {A1},k} + \beta _{\text {A2}} \text {Condition}_{\text {A2},k} + \beta _{\text {B}} \text {Condition}_{\text {B},k} \nonumber \\&+ \beta _{\text {A1,Trial}} \text {Trial}_{l} \text {Condition}_{\text {A1},k} + \beta _{\text {A2,Trial}} \text {Trial}_{l} \text {Condition}_{\text {A2},k} + \beta _{\text {B,Trial}} \text {Trial}_{l} \text {Condition}_{\text {B},k} \nonumber \\&+ b_{\textrm{Pair},m} + \varepsilon _{klm} . \end{aligned}$$

**Fig. 2:**
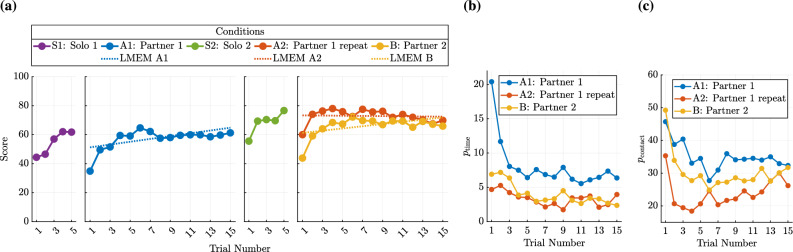
Development of the performance score and its underlying penalties over the trial progression in different conditions. **(a)** Mean of the performance score is shown over the trial numbers within the block condition of first solo (S1), collaborative training (A1), second solo (S2), repetition with the previous partner (A2), and switching to an unfamiliar partner (B). The dotted lines show the change of the score in the different collaboration conditions according to the LMEM of Eq. (6). The scores in the first trial of the collaboration conditions are captured by $\beta _\textrm{A1}$, $\beta _\textrm{A2}$, $\beta _\textrm{B}$, while score changes over trials are represented by $\beta _\textrm{A1, Trial}$, $\beta _\textrm{A2, Trial}$, $\beta _\textrm{B, Trial}$. The development of the average time penalty **(b)** and the contact penalty **(c)** over the trials in the different collaboration conditions (A1, A2, B) is shown, indicating their influence on the performance score.

Here, $\text {Score}_{klm}$ is the performance score in the *k*-th condition ($k \in \{ 1, 2, 3 \}$) for the *l*-th trial ($l \in \{ 1, \ldots , 15 \}$) of the *m*-th pair of participants ($m \in \{ 1, \ldots , 44 \}$). The categorical fixed effect $\text {Condition}_{\textrm{Cond},k}$ indicates the collaboration condition, where $\textrm{Cond} \in \{\text {A1}, \text {A2}, \text {B}\}$ corresponds to conditions A1 (Partner 1), A2 (Partner 1 repeat), and B (Partner 2). These categorical variables are equal to 1 if the observation belongs to the respective condition and 0 otherwise. The fixed effect coefficients $\beta _{\text {A1}}, \beta _{\text {A2}}$, and $\beta _{\text {B}}$ represent the estimated score in the first trial of each condition. The term $\text {Trial}_{l} \in \{0, \ldots ,14\}$ denotes the trial number and the coefficients $\beta _{\text {A1,Trial}}, \beta _{\text {A2,Trial}}, \beta _{\text {B,Trial}}$ capture how the score changes over the trial number in each collaboration condition. The random effect $b_{\textrm{Pair},m} \sim \mathcal {N}(0, \sigma _{\textrm{Pair}}^2)$ accounts for differences in performance levels between pairs, with its variances $\sigma _{\textrm{Pair}}^2$ estimated by the LMEM. Finally, the residual term $\varepsilon _{klm} \sim \mathcal {N}(0, \sigma _\varepsilon ^2)$ captures the remaining unexplained variation of observations. Detailed results for this LMEM are presented in Supplementary Table S1. The condition-specific fixed effects were statistically compared within the model using pairwise linear contrasts evaluated with t-tests, and the resulting p-values were adjusted for multiple comparisons using Bonferroni-Holm correction^34^. Results from this comparison indicate that repeating collaboration with the same partner in condition A2 resulted in significantly higher initial performance compared to training with this partner (A1) ($p < 0.001$, $\beta _\textrm{A2} > \beta _\textrm{A1}$). This is reflected by the estimated score in the first trial of condition A2 ($\beta _\textrm{A2}$) exceeding that of condition A1 ($\beta _\textrm{A1}$). When comparing the estimated score for the first trial of training with a partner in A1 ($\beta _\textrm{A1}$) to the estimated initial score when switching to an unfamiliar partner in condition B ($\beta _\textrm{B}$), no significant difference was found ($p = 0.062$, $\beta _\textrm{A1}$ vs. $\beta _\textrm{B}$). However, switching to an unfamiliar partner (B) led to a lower initial performance than in A2 ($p = 0.037$, $\beta _\textrm{B} < \beta _\textrm{A2}$). In Fig. 2(a), this difference in initial performance between collaboration with an unfamiliar or familiar partner is shown by the red and yellow regression lines starting with different score values at the first trial. This highlights that familiarity with a partner resulted in higher initial performance. The performance change over trials also differed depending on the familiarity with the partner. During the initial collaboration condition (A1), performance improved significantly across trials ($p < 0.001$, $\beta _\textrm{A1, Trial} > 0$), which is captured by the coefficient $\beta _\textrm{A1, Trial}$. This trend is shown by the positive slope of the blue regression line in Fig. 2(a). With the unfamiliar partner (B) a similar performance improvement as in condition A1 ($p=0.37$, $\beta _\textrm{B, Trial}$ vs. $\beta _\textrm{A1, Trial}$) was observed. However, repeating collaboration with the known partner (A2) showed no significant performance improvement over trials ($p = 0.70$, $\beta _\textrm{A2, Trial}$), suggesting a performance plateau^35^, possibly due to the effects of mental or physical fatigue^36^. An analysis using a simplified version of the LMEM from Eq. (6), was conducted to compare performance between conditions A2 and B at a specific trial. In this simplified LMEMs, separate models were fitted for each trial number, excluding the trial number as a model predictor. The results revealed that from the fourth trial onwards, the difference in performance between these two conditions became non-significant ($p>0.13$, $\beta _\textrm{B}$ vs. $\beta _\textrm{A2}$). This indicates that while performance was initially lower with an unfamiliar partner, repeated collaboration enabled participants to rapidly adapt to the new partner, allowing the new pairs to achieve the same performance level as the familiarized participant pairings. These findings suggest the development of partner-specific collaboration behavior, which enables immediate high performance with a familiar partner while requiring re-negotiation of the collaboration behavior when interacting with an unfamiliar partner (H1).

Because we are mainly interested in how partner familiarity affects overall task performance, the trends of time and contact penalties are described qualitatively to provide additional context for the statistically evaluated score results. Figure 2(b) shows that the time penalty was initially highest in the training condition (A1) and decreased most in the initial three trials in this condition. For conditions A2 and B, the time penalty started at a similar level, showing only a slight decrease over trials. Since the time penalty primarily improved during initial collaboration (A1), this suggests that its improvement was mainly driven by task learning and relatively unaffected by familiarity with the partner. Because the contact penalty, shown in Fig. 2(c), was generally on a larger scale than the time penalty, it had a bigger influence on the score values. The contact penalties in condition B resembled those in condition A1, starting high and decreasing over trials. In A2, however, the contact penalty began lower, initially declined, but then increased, approaching levels seen in A1 and B. This trend indicates that prolonged interaction may have led to fatigue or other factors that limit achievable motion accuracy. These observations suggest that contact penalties were likely more influenced by familiarity with the partner than time penalties, because joint motion accuracy may rely more on precise coordination of individual contributions (H1).

**Fig. 3:**
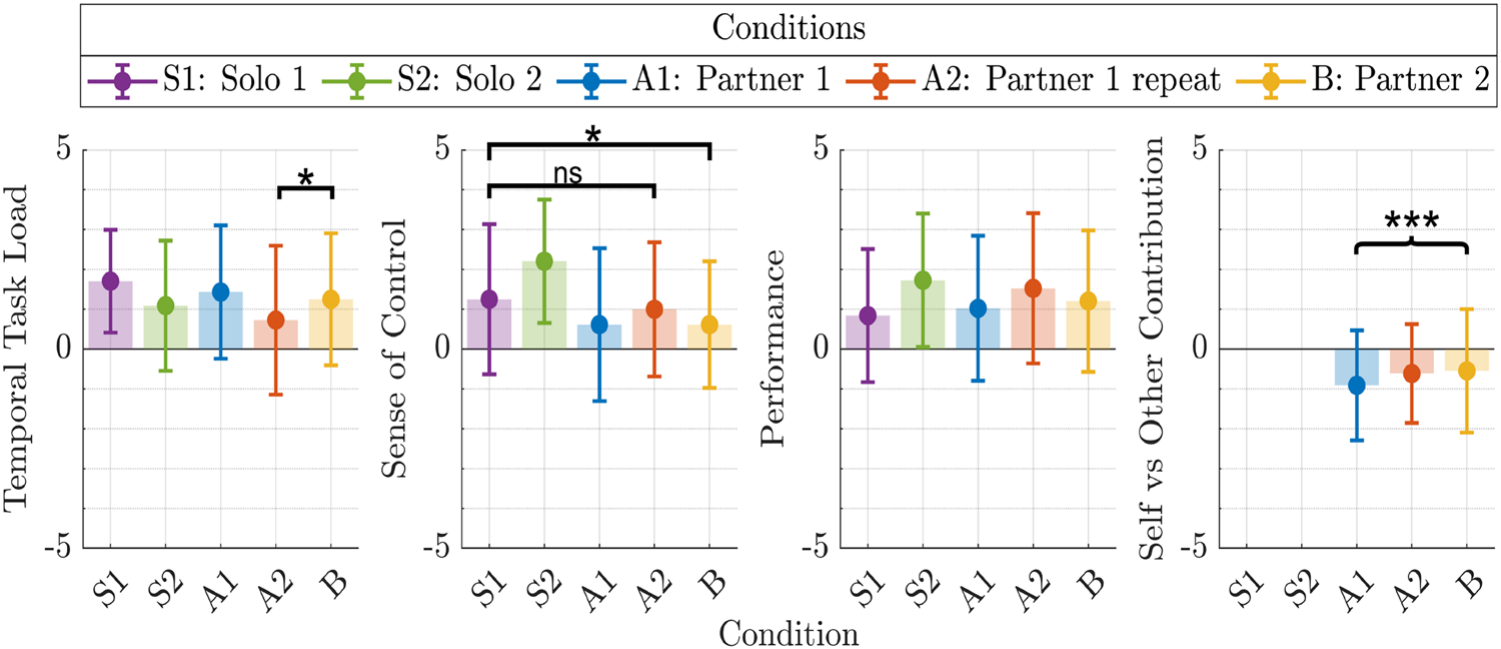
Mean and standard deviation of the subjective ratings for temporal task load, sense of control, subjective performance, and contribution in the different experiment block conditions.

After examining the effect of partner familiarity on performance scores, we investigate the influence of familiarity with the partner on the subjective assessment of collaboration. The subjective ratings of temporal task load, subjective performance, sense of control, and contribution are shown in Fig. 3. Comparisons between the conditions were made directly within the following LMEM using pairwise linear contrasts of the estimated fixed effects evaluated with t-tests:

$$\begin{aligned} \text {Rating}_{\hat{k}n} = \beta _{\text {S1}} \text {Condition}_{\text {S1},\hat{k}} + \beta _{\text {A1}} \text {Condition}_{\text {A1},\hat{k}} + \beta _{\text {A2}} \text {Condition}_{\text {A2},\hat{k}} + \beta _{\text {B}} \text {Condition}_{\text {B},\hat{k}} + b_{\textrm{Participant},n} + \varepsilon _{\hat{k}n} . \end{aligned}$$

$\text {Rating}_{\hat{k}n}$ represents the subjective rating in $\hat{k}$-th condition ($\hat{k} \in \{ 1, \ldots , 4 \}$) from the *n*-th participant ($n \in \{ 1, \ldots , 44 \}$). The categorical fixed effect $\text {Condition}_{\textrm{Cond},\hat{k}}$, where $\textrm{Cond} \in \{\text {S1}, \text {A1}, \text {A2}, \text {B}\}$, indicates the block condition, with $\beta _{\text {S1}}, \beta _{\text {A1}}, \beta _{\text {A2}}, \beta _{\text {B}}$ reflecting the effect of conditions on the subjective rating. The random effect $b_{\textrm{Participant},n}$ captures participant-specific differences in subjective ratings, and $\varepsilon _{\hat{k}n}$ is the residual error. Participants reported feeling more rushed when collaborating with an unfamiliar partner (B) rather than with a familiar partner (A2) ($p=0.049$, $\beta _\textrm{B} > \beta _\textrm{A2}$). Comparing the sense of control in conditions A2 and B to the one from the first solo condition (S1) shows a familiar partner (A2) did not diminish the sense of control compared to performing the task alone ($p=0.39$, $\beta _\textrm{A2}$ vs. $\beta _\textrm{S1}$). Conversely, with an unfamiliar partner (B), the sense of control was reduced ($p=0.031$ , $\beta _\textrm{B} < \beta _\textrm{S1}$). This result, along with the reduction in perceived time pressure when collaborating with a familiar partner, suggests that working with an unfamiliar partner could have imposed additional cognitive demands (H1). The relationship between participants’ sense of control and their collaboration performance was analyzed using the following LMEM, which accounts for performance variability at both the participant and condition levels:

$$\begin{aligned} \text {Performance}_{kn} = \beta _0 + \beta _{\text {Control}} \text {Control}_{kn} + b_{\textrm{Participant},n} + b_{\textrm{Condition},k} + \varepsilon _{kn} . \end{aligned}$$

Here, $\text {Performance}_{kn}$ represents either the subjective performance rating or the objective task score for the *k*-th collaboration condition and for the *n*-th participant. $\text {Control}$ denotes the participant’s sense of control rating, with coefficient $\beta _{\text {Control}}$ reflecting the relationship between sense of control and performance. $\beta _0$ represents the estimated performance when the sense of control rating is zero. The random effect $b_{\textrm{Participant},n}$ accounts for participant-specific performance differences, and $b_{\textrm{Condition}, k}$ captures performance variability across conditions. The residual error is denoted $\varepsilon _{kn}$. Results showed that a higher sense of control was associated with both better subjective performance ratings ($\beta _\textrm{Control} = 0.65$, $SE = 0.073$, $t(130) = 8.84$, $p < 0.001$) and higher objective task scores ($\beta _\textrm{Control} = 3.10$, $SE = 0.85$, $t(130) = 3.67$, $p < 0.001$) during collaboration. The standard error *SE* indicates how strongly the estimated coefficient $\beta$ varies. The significance of the coefficient is assessed using the t-statistic *t*(*df*) with the associated degrees of freedom *df*, which represent the amount of information available for estimating the coefficient. The t-distribution determined by *df* is used to decide whether the t-statistic is large enough to indicate a statistically significant result. These results highlight the importance of the sense of control, and therefore the familiarity with the partner, for effective collaboration (H1). The subjective ratings from participant’s questionnaires further show that participants consistently rated their own contributions during collaboration as lower than those of their partners. This was confirmed by a one-sample one-tailed t-test for subjective rating of contribution across conditions A1, A2, and B1 ($p < 0.001$).

**H1:** In summary, these findings underscore the role of partner familiarity in facilitating effective collaboration in terms of collaboration performance and subjective assessment of the collaboration. This is supported by higher initial performance with a familiar partner (A2 > A1, $p < 0.001$; A2 > B, $p = 0.037$), and a reduced sense of control and increased subjective time pressure when collaborating with an unfamiliar partner ($p = 0.025$ and $p = 0.049$, respectively).

**Fig. 4:**
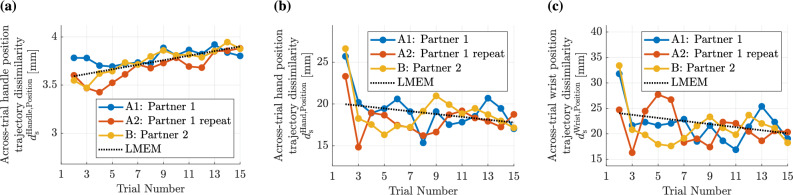
Developemnt of consecutive motion dissimilarity over trials. Consecutive motion dissimilarity metrics are shown for handle position trajectories **(a)**, hand position trajectories **(b)**, and wrist position trajectories **(c)**. The trend over trials, estimated using the LMEM with Eq. (7), is displayed as a black-dotted line, which starts for the second trial at $\beta _0$ and changes with slope $\beta _\textrm{Trial}$ over the trial number.

### (H2) Action predictability during high-precision collaboration

We aimed to investigate whether participants learned to reduce the variability of their motions across trials to improve collaboration performance. Figure 4 illustrates how the dissimilarity of motions from consecutive trials $d_{\textrm{s}}$ changed over the trial number in the collaboration block conditions. Specifically, we analyzed the dissimilarities of consecutive handle $d_{\textrm{s}}^\textrm{Handle, Position}$, hand $d_{\textrm{s}}^\textrm{Hand, Position}$, and wrist $d_{\textrm{s}}^\textrm{Wrist, Position}$ position trajectories. Because two participants collaborated, two consecutive motion dissimilarity metrics of hand and wrist movements, calculated according to Eq. (5), are available for all collaboration trials. Therefore, for the hand and wrist motion dissimilarity, the metric values of the partners were averaged in each trial to obtain a single dissimilarity metric per body part for each trial. The change of dissimilarity over trials was analyzed considering its variability between pairs and conditions with a LMEM of the form:

7 $$\begin{aligned} d_{\textrm{s},k\hat{l}m} = \beta _0 + \beta _{\text {Trial}} \text {Trial}_{\hat{l}} + b_{\textrm{Pair},m} + b_{\textrm{Condition},k} + \varepsilon _{k\hat{l}m}. \end{aligned}$$

**Fig. 5:**
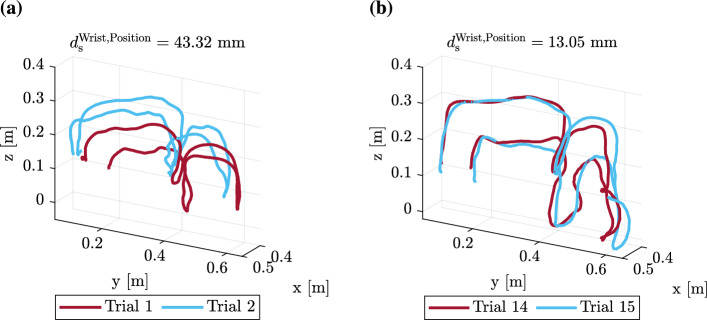
Progression towards consistent wrist movements during repeated collaboration. Exemplary wrist motion trajectories from one participant during the training collaboration condition (A1) show the greater dissimilarity in **(a)** the initial trials ($d_\textrm{s}^\textrm{Wrist, Position} = {43.32}\,{\hbox {mm}}$) compared to **(b)** the final trials ($d_\textrm{s}^\textrm{Wrist, Position} = {13.05}\,{\hbox {mm}}$), demonstrating increased consistency in wrist movements as collaboration trials progress.

Here, $d_{\textrm{s}, k\hat{l}m}$ represents the dissimilarity of subsequent motions in the *k*-th condition for the $\hat{l}$-th trial ($\hat{l} \in \{ 1, \ldots , 14 \}$) of the *m*-th pair. The term $\text {Trial}_{\hat{l}} \in \{0, \ldots ,13\}$ denotes the trial number, with coefficient $\beta _{\text {Trial}}$ reflecting the change in dissimilarity over trials. The coefficient $\beta _0$ represents the estimated dissimilarity level without the effect of trials. The random effects $b_{\textrm{Pair},m}$ and $b_{\textrm{Condition},k}$ capture the variability of the dissimilarity metric across pairs and experimental conditions, and $\varepsilon _{k\hat{l}m}$ represents the residual error. For example, the dissimilarity between the wrist position trajectory from the second and the first trial in condition A1 is calculated as $d(\mathbf{X}_{\text {Wrist}, A1, 2}, \mathbf{X}_{\text {Wrist}, A1, 1})$, where $\mathbf{X}_{\textrm{Wrist,A1},\hat{l}} \in \mathbb {R}^{N_{\hat{l}} \times 3}$ is the 3D wrist position trajectory from the $\hat{l}$-th trial in condition A1 with $N_{\hat{l}}$ samples. The results of the LMEM revealed a significant decrease in the dissimilarity of consecutive arm movements for the hand and wrist ($p<0.01$, $\beta _\textrm{Trial}^\textrm{Hand,Position} < 0$, $\beta _\textrm{Trial}^\textrm{Wrist,Position} < 0$), as shown by the negative slope of the regression lines in Fig. 4(b, c). The detailed results are presented in Supplementary Table S2. This suggests that participants may converge to consistent arm movements over time (H2). In contrast, the trajectories of the handle positions became increasingly dissimilar ($p<0.001$, $\beta _\textrm{Trial}^\textrm{Handle,Position} > 0$), which may be due to the increasing difficulty in precisely controlling the handle as the trial progressed. Figure 5(a, b) illustrates the difference between high and low consecutive motion dissimilarity, exemplified by one participant’s initial and final wrist motions.

A decrease in inter-trial variability of motions suggests improved predictability, which could affect collaboration performance. To verify this hypothesis statistically, the LMEM from Eq. (6) is extended by the dissimilarity metric $d_{\textrm{s}, k\hat{l}m}$ as an additional fixed effect with the associated coefficient $\beta _{\textrm{s}}$. Since the dissimilarity measure compares motions between consecutive trials within the conditions, there is one less observation per condition than the number of trials. To ensure consistency, the first trial of each condition is excluded from the LMEM in Eq. (6). This model is chosen because it allows for the examination of how motion dissimilarity influences performance while accounting for the effects of trial progression, condition differences, and inherent pair-specific score variability. The dissimilarity metrics of the handle, hand, or wrist motions are added in separate models. All consecutive trial dissimilarity metrics significantly lowered the performance during collaboration ($p<0.005$, $\beta _{\textrm{s}} < 0$). In other words, the more similar consecutive motions were, the higher the achieved performances were (H2). This is especially noticeable for the dissimilarity of consecutive handle motions with its comparatively high influence on the score (see Supplementary Table S3). Because of the task’s high precision requirements for the handle, the consecutive handle motion dissimilarity was on a smaller scale than that of the arm motions, which were further away from the wire path to be tracked. The comparatively smaller scale of the handle dissimilarity metric values could explain their higher impact on the score. We also saw that the dissimilarity of the handle position trajectories increased over the course of the trials. This suggests that the increase in the dissimilarity of consecutive handle movements may not have been due to intentional collaboration behavior, such as task exploration^37^. Instead, it was probably caused by unwanted side effects such as fatigue^36^. As the number of trials increases, the continuously high precision requirements could make it difficult for participants to maintain high accuracy over longer periods of time. Since the hand and wrist motions could have been adjusted without changing the handle positioning relative to the wire path, they may have converged to motions that were more ergonomic without impairing the task performance.

**H2:** In summary, while not all movements showed a consecutive reduction in variability, performing repetitive and predictable motions significantly improved collaboration performance, likely due to the increased predictability of the motions. This is supported by a high consecutive-motion dissimilarity significantly decreasing performance for all tested motion components ($p < 0.005$).

**Fig. 6:**
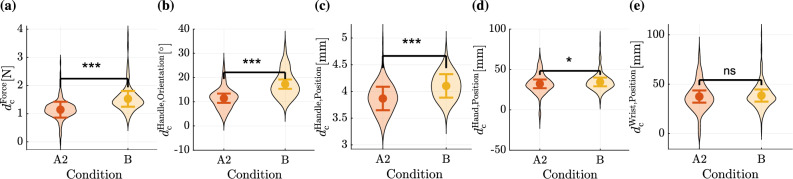
Similarity of motion and force time series from condition A2 (Partner 1 repeat) or B (Partner 2) to the final trial of condition A1 (Partner 1). Each plot illustrates the estimated fixed effect coefficients of this dissimilarity metric $d_\textrm{c}$ for the two conditions A2 and B along with their 95% confidence intervals. Additionally, the distributions of the metric data are visualized through violin plots. The force profiles **(a)**, handle orientation trajectories **(b)**, handle position trajectories **(c)**, and hand position trajectories **(d)** were significantly more similar to the training condition A1 in condition A2 than in condition B. Wrist position trajectories **(e)** did not showed significantly different $d_\textrm{c}$ values in condition A2 than in condition B.

### (H3) Partner-specifc interaction dynamics

During the collaboration, participants may adapt their collaboration behaviors, such as interaction dynamics (i.e., motions and forces), to their specific partner to optimize the collaboration. Therefore, we investigate if the interaction dynamics established during the training (A1) are specific to the training partner. This is analyzed by examining how similar the movements and forces at the end of the training (A1) are to those when the collaboration is either repeated with the familiar partner (A2) or with an unfamiliar partner (B). This training dissimilarity $d_\textrm{c}$ is computed using Eq. (5). Specifically, we considered the training dissimilarity of force $d_{\textrm{c}}^\textrm{Force}$, handle orientation $d_{\textrm{c}}^\textrm{Handle, Orientation}$, handle position $d_{\textrm{c}}^\textrm{Handle, Orientation}$, hand position $d_{\textrm{c}}^\textrm{Hand, Position}$, and wrist position $d_{\textrm{c}}^\textrm{Wrist, Position}$ trajectories. For example, the training dissimilarity of the wrist position trajectory from the *l*-th trial of condition A2 is calculated as $d(\mathbf{X}_{\text {Wrist}, \text {A1}, 15}, \mathbf{X}_{\text {Wrist}, \text {A2}, l})$. To analyze the effect of conditions A2 and B on the training dissimilarity $d_\textrm{c}$ while accounting for pair-level variability of training dissimilarity, we use the LMEM:

8 $$\begin{aligned} d_{\textrm{c},\tilde{k}lm} = \beta _{\text {A2}} \text {Condition}_{\text {A2},\tilde{k}} + \beta _{\text {B}} \text {Condition}_{\text {B},\tilde{k}} + b_{\textrm{Pair},m} + \varepsilon _{\tilde{k}lm}. \end{aligned}$$

Here, $d_{\textrm{c},\tilde{k}lm}$ represents the training dissimilarity for the $\tilde{k}$-th condition ($\tilde{k} \in \{ 1,2 \}$) in the *l*-th trial of the *m*-th pair. The categorical fixed effect $\text {Condition}_{\textrm{Cond}, \tilde{k}}$, where $\textrm{Cond} \in \{\text {A2}, \text {B}\}$, indicates the experimental condition, with the coefficients $\beta _{\text {A2}}$ and $\beta _{\text {B}}$ capturing the effect of conditions on the training dissimilarity. The random effect $b_{\textrm{Pair},m}$ accounts for pair-specific differences in the dissimilarity metric, and $\varepsilon _{\tilde{k}lm}$ is the residual error. The results are visualized in Fig. 6 for different motions and the interaction force. Detailed results of the analysis with the LMEM are listed in Supplementary Table S4. The estimated fixed effect coefficients of conditions A2 and B were statistically compared within the LMEM through a pairwise linear contrast using a t-test. The trajectories of the handle position ($p<0.001$), the handle orientation in Euler angles ($p<0.001$), the hand position ($p=0.027$), and the forces ($p<0.001$) from the last trial of condition A1 were more similar to those from condition A2 than to those from condition B ($\beta _\textrm{A2} < \beta _\textrm{B}$). However, wrist position trajectories did not show significant differences in training dissimilarity between conditions A2 and B ($p=0.41$, $\beta _\textrm{A2}^\textrm{Wrist, Position}$ vs. $\beta _\textrm{B}^\textrm{Wrist, Position}$). As the distance from the manipulation target (i.e., the wire path) increased, the differences in training dissimilarities between conditions A2 and B became less significant, suggesting that these movements may have been less influenced by the specific partner. Instead, these movements of the hand and especially the wrist may have been adjusted based on other factors like ergonomic aspects^38^.

**H3:** In summary, these findings suggest that interaction dynamics were partner-specific, as the participants used different interaction dynamics when working with an unfamiliar partner than with a familiar partner. Participants recalled the interaction dynamics established during training (A1) when collaborating with the same partner (A2), as evidenced by significantly lower training dissimilarities in A2 compared to B for forces, handle orientation, handle position, and hand position trajectories ($p < 0.001$, $p < 0.001$, $p < 0.001$, and $p = 0.027$, respectively).

### (H4) Effects of partner differences on performance and learning capabilities

We analyze the effect of interpersonal differences between partners on collaboration. First, the individual performance levels are considered. The influence of individual performance differences on the improvement in individual performance resulting from collaboration was analyzed using the linear model:

9 $$\begin{aligned} \Delta \text {Score}_{S2-S1,n} = \beta _0 + \beta _{\Delta \text {S1, Self-Other}} \Delta \text {Score}_{S1,\mathrm{Self - Other},n} + \varepsilon _{n}. \end{aligned}$$

**Fig. 7:**
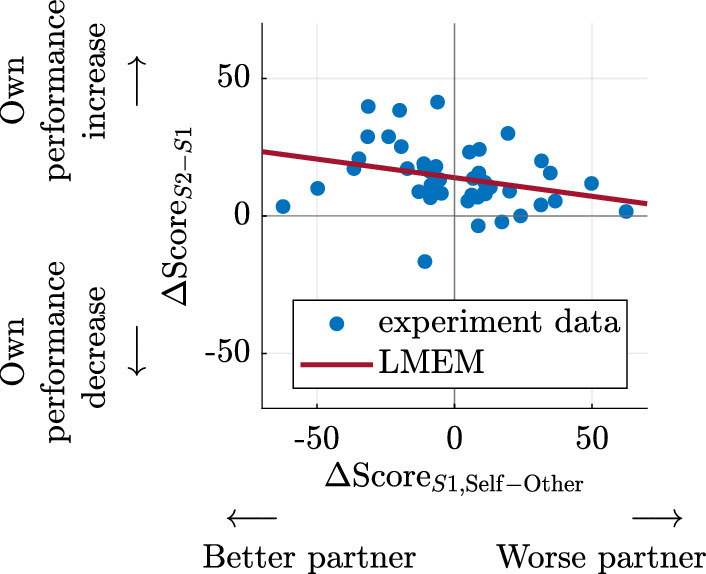
Correlation of the individual performance difference between partners with the individual score improvement after collaboration. The individual performance difference $\Delta \textrm{Score}_\mathrm{S1, Self-Other}$ was computed as the difference in score between the partnered individuals during the first solo task execution (S1). Individual performance improvement $\Delta \textrm{Score}_\mathrm{S2-S1}$ was determined as the increase in individual score from the first (S1) to the second (S2) solo task execution. The red line with a slope of $\beta _{\Delta \text {S1, Self-Other}}$ represents the modeled relationship based on the LMEM with Eq. (9).

Here, $\Delta \text {Score}_{S2-S1,n}$ represents the change in average score from the first solo block (S1) to the second solo block (S2) for the *n*-th participant. The fixed effect $\Delta \text {Score}_{S1,\mathrm{Self - Other}, n}$ is the average performance difference between the paired individuals during the first solo block, with coefficient $\beta _{\Delta \text {S1, Self-Other}}$ capturing its influence on the individual performance improvement. $\beta _0$ reflects the individual performance improvement in case of no performance difference between partners, and $\varepsilon _{n}$ is the residual error. This model allows for an assessment of how performance differences between collaborators during the first solo block (S1) influence the individual improvement from S1 to the second solo block (S2). Participants who performed worse than their partners in the first solo block (S1) showed greater improvement from S1 to the second solo block (S2) ($\beta _{\Delta \text {S1, Self-Other}} = -0.14$, $SE = 0.07$, $t(42) = -2.03$, $p=0.048$). Conversely, participants who performed better than their partners in S1 showed less performance improvement from S1 to S2. This is illustrated by the negative slope of the LMEM’s regression line in Fig. 7. This finding indicates that performance transfer between participants may depend on the difference in the individual performance levels of the two partners (H4). We further observed that larger individual performance differences between collaborators decreased the performance during collaboration ($\beta _{| \Delta \textrm{S1, Pair} |} = -0.39$, $SE = 0.16$, $t(915) = -2.45$, $p = 0.015$). This was determined using an extension of the LMEM from Eq. (6) that incorporates the absolute difference in scores between the two partners during the first solo block (S1) $| \Delta \textrm{Score}_{S1,\textrm{Pair}} |_{m}$ as an additional fixed effect, with its associated coefficient $\beta _{| \Delta \textrm{S1, Pair} |}$. This allows the model to examine how individual performance differences between collaborators during the first solo block (S1) influence the collaboration score $\text {Score}_{klm}$ in addition to the effects of the LMEM with Eq. (6). A greater disparity in individual performance may, therefore, hinder collaborative performance (H4).

Next, we considered how differences in partner characteristics influenced collaboration performance. The characteristics considered were anthropometric variables, body height and weight, but also other features such as age, handedness (left, right, ambidextrous), and gender (male, female, other). The effect on collaboration performance was analyzed by extending the LMEM from Eq. (6) with an additional fixed effect $\Delta _{\textrm{char},m}$, representing the differences in a specific characteristic (e.g. age) between partners of the *m*-th pair. The coefficient $\beta _{\Delta \textrm{char}}$ captures the effect of these differences in characteristics on collaboration performance. For gender and handedness, this fixed effect is a binary variable that indicates whether the partners differ in this characteristic ($\Delta _{\textrm{char},m} = 1$) or do not differ ($\Delta _{\textrm{char},m} = 0$). Separate extended LMEMs were created for each characteristic to examine its influence on the collaboration score $\text {Score}_{klm}$, while retaining the original fixed effects for trial progression and condition differences, as well as the pair-specific differences in score. A difference in handedness between participants significantly decreased performance ($\beta _{\Delta \textrm{char}}^\textrm{handedness} = -13.71$, $SE = 5.56$, $t(915) = -2.46$, $p = 0.014$). This indicates that pairs in which both partners are left- or right-handed may have achieved higher scores (H4). Given the imbalance in handedness among participants in our experiment (more right-handed than left-handed), we conducted a stratified bootstrapping analysis with 1,000 iterations to assess the robustness of the observed effect of differing handedness on performance. This method preserved the original proportion of handedness differences in each resampled dataset by resampling within groups defined by same or differing handedness^39^. While bootstrapping does not fully overcome the uneven handedness distribution and the small number of mixed-handed pairs, it provides a useful assessment of the effect’s robustness. The stratified bootstrapped $95\%$ confidence interval for the effect of differing handedness was $[ -25.06, -1.26 ]$, confirming that its negative impact on collaboration performance remains significant despite the data imbalance. Other differences in partner characteristics did not affect the collaborative performance ($p > 0.4$, $\beta _{\Delta \textrm{char}}$). We investigated if the reduced collaboration performance of mix-handed pairs is caused by the task being more challenging to left-handed participants since they are required to execute the task with their non-dominant hand. The effect of handedness on solo task performance was analyzed using the LMEM

10 $$\begin{aligned} \text {Score}_{\breve{k}\breve{l}n} = \beta _\textrm{right} \text {Hand}_{\textrm{right}, n} + \beta _\textrm{left} \text {Hand}_{\textrm{left}, n} + \beta _\textrm{amb} \text {Hand}_{\textrm{amb}, n} + b_{\textrm{Condition}, \breve{k}} + b_{\textrm{Participant}, n} + \varepsilon _{\breve{k}\breve{l}n}. \end{aligned}$$

Here, $\text {Score}_{\breve{k}ln}$ represents the score in the $\breve{k}$-th solo condition ($\breve{k} \in \{1, 2\}$) for the $\breve{l}$-th trial ($\breve{l} \in \{1,\dots ,5\}$) of the *n*-th participant. The categorical fixed effects $\text {Hand}_{\text {right},n}$, $\text {Hand}_{\text {left},n}$, and $\text {Hand}_{\text {amb},n}$ indicate the handedness of the *n*-th participant (right-handed, left-handed, ambidextrous), with the corresponding coefficients $\beta _\textrm{right}$, $\beta _\textrm{left}$, and $\beta _\textrm{amb}$ capturing the effect of each handedness type on the task score. The random effects $b_{\textrm{Condition}, \breve{k}}$ and $b_{\textrm{Participant}, n}$ account for score differences between solo conditions and participants, while $\varepsilon _{\breve{k}\breve{l}m}$ is the residual error term. The estimated fixed effect coefficients of the handedness types were statistically compared within the LMEM using pairwise linear contrasts evaluated with t-tests, and the resulting p-values were adjusted for multiple comparisons using the Bonferroni-Holm correction. The results of this analysis revealed no statistically significant differences in solo task performance between participants depending on their handedness ($p>0.6$, $\beta _\textrm{right}$ vs. $\beta _\textrm{left}$ vs. $\beta _\textrm{amb}$). Detailed results for this LMEM are presented in Supplementary Table S5.

Finally, we assessed whether differences in partner characteristics affect individual learning with the linear model:

$$\begin{aligned} \Delta \text {Score}_{S2-S1,n} = \beta _0 + \beta _{\mathrm{S2-S1},\Delta \text {char}} \Delta \text {char}_{n} + \varepsilon _{n}. \end{aligned}$$

The fixed effect $\Delta \text {char}_{n}$ denotes the difference in a characteristic between the *n*-th participant and the corresponding partner in collaboration condition A1, with $\beta _{\mathrm{S2-S1},\Delta \text {char}}$ capturing its effect on individual performance improvement. $\beta _0$ accounts for this individual improvement in case of no difference of characteristic between partners, and $\varepsilon _{n}$ is the residual error. None of the differences in partner characteristics affected individual learning through collaboration ($p>0.16$, $\beta _{\mathrm{S2-S1},\Delta \text {char}}$) (H4).

**H4:** In summary, these results suggest that interpersonal differences in individual performance levels and handedness can influence physical collaboration performance and individual learning capabilities, emphasizing the importance of considering partner compatibility in collaboration tasks. Specifically, larger individual performance differences of partners led to greater individual learning gains for the worse-performing partner ($p=0.048$) but reduced collaborative performance ($p=0.015$). Differing handedness between paired individuals significantly lowered collaborative task scores ($p=0.014$).

## Discussion

In this study, we investigated the learning behavior of haptically coupled collaborators in a task with high precision demands. We examined which collaboration behaviors are adopted over repeated interactions, whether these learned collaboration behaviors are specific to the partner, and how interpersonal differences influence collaboration.

Improvements in performance in the hot wire task can be attributed to different factors. For example, one potential factor is practicing the task itself, e.g. learning the course of the wire through self-induced action effects such as moving and touching the wire^40^. Another possible factor is learning to better predict the partner’s actions and their effects^6,7^. The results from our study indicate that by repeating physical collaboration in a high-precision task with the same partner, learned collaboration behaviors are effectively retained and utilized to enhance collaboration performance. This observation may indicate that a degree of familiarization with the partner’s influence occurred with prolonged practice, as knowledge about the collaborator’s behavior is learned and used to enhance interpersonal coordination. When switching to an unfamiliar partner, the transfer of learned collaboration knowledge was limited, because a similar level of performance as with a familiar partner was only achieved after a retraining phase with the unfamiliar partner, as theorized in a previous work^13^. These findings indicate that learned behaviors are at least partially partner-specific (**H1**) , which may provide initial benefits during repeated collaboration with a familiar partner. When switching to an unfamiliar partner, participants reported a reduction in the sense of control and increased time pressure compared to executing the task with a familiar partner, suggesting additional subjective cognitive demands with an unfamiliar partner. When switching to an unfamiliar partner, any potentially learned partner-specific predictive model will inherently result in a higher prediction error, as it does not account for the unfamiliar partner’s collaboration behavior. Consequently, this model needs to be retrained or adapted to accommodate the new collaboration dynamics. Although a prior study examined how varying levels of partner expertise influence physical collaboration performance with an unfamiliar partner^28^, our study examines a different aspect by investigating if learned collaboration knowledge is specific to the partner and how switching to an unfamiliar partner affects collaboration.

We observed that during collaboration the partner contribution was subjectively higher-rated. One possible factor influencing this perception can be the force escalation/sensory attenuation effect, where self-generated stimuli, like forces, are perceived to be lower than external stimuli^41^. Sensory attenuation has been shown to facilitate self-other distinction through temporal cues in an auditory joint action task^42^. Research on human collaboration has emphasized the importance of self-other distinction and integration in joint actions^9,11^. In our physical collaboration task sensory attenuation may enhance sensitivity to haptic information from the partner. This could improve the ability to distinguish between self-generated and partner-induced action effects, allowing individuals to adjust their actions more accurately in response to their partner’s contributions, which ultimately leads to better coordination. Further investigation is required to understand the role of sensory attenuation in haptic communication during physical collaboration.

Collaborators converged to a stereotypical arm motion trajectory indicated by reduced inter-trial variability. Lower inter-trial variability of motions allowed for higher collaboration performance, which could be explained by the increase in action predictability (**H2**). While previous studies have shown that learning to reduce action variability can enhance predictability and performance in both non-physical and physical collaboration tasks^14–18^, our findings extend these results to a physical task with high-precision requirements. This demonstrates that the reduction of joint action variability is a collaboration strategy employed across a wide range of collaborative tasks. Through the repeated experience of the partner’s action effects, a predictive model^6,7^ of the partner’s task contribution may be built and refined. This predictive model could be used to progressively improve the coordination of one’s own actions with those of the partner, allowing to reduce variability and consequently increase the predictability of joint actions.

When collaborators repeated the collaboration with a familiar partner, their movements and forces showed high similarity to those observed during initial training with that partner. In contrast, switching to an unfamiliar partner resulted in noticeably different interaction dynamics, reflected by motion and force patterns that differed from those with the initial partner. This indicates that the interaction dynamics established with a collaboration partner were specific to the partner (**H3**). The partner-specific interaction dynamics were effectively retained^19,20^ in subsequent collaborations with a known partner. This retention of learned interaction dynamics likely contributes to the higher initial performance observed with familiar partners, as the established interpersonal coordination can be recalled without the need for re-negotiation^13^. Conversely, collaborating with an unfamiliar partner likely requires establishing new interaction behaviors. However, after a brief retraining phase of a few trials, performance with an unfamiliar partner can match the one with a familiar partner. This suggests that the interaction dynamics learned from collaboration with one partner could be used to enable fast adjustment to new collaborators. Retention in physical cooperation tasks has already been investigated in a previous study^22^. However, our findings extend those results by showing that during physical collaboration partner-specific interaction dynamics are used, which can be recalled in subsequent collaborations with a familiar partner.

Our findings indicate that certain partner differences can significantly affect both individual performance improvement and collaborative outcomes (**H4**). Specifically, individual performance differences between partners lowered the collaboration performance, aligning more with the findings of a previous study^24^. Contrasting findings were reported in other works^1,25^. Furthermore, individuals who initially performed worse than their partners exhibited greater improvements after collaboration, indicating a transfer of skill or knowledge from the higher-performing participants to their partners. The enhanced knowledge transfer with a better-performing partner could be caused by self-other integration, as individuals integrate their partner’s superior task strategies into their own behavior. This finding contrasts with previous results^25,28^, where individual performance improved more when collaborating with a partner of similar skill level. When individuals were paired with novices instead of experts, individuals were able to collect more experience with the task as more exploration of task dynamics occurred^28^. In our hot wire task, however, exploration was limited due to the high-precision requirements, which could explain the different findings. Additionally, all participants in our experiment were novices who had no prior practice with the task, limiting the observed range of expertise in participants. It is possible that knowledge transfer is diminished when performance differences are larger. When novices collaborate with highly skilled partners, they may assume a more passive role, thus engaging less with the task. The reduced engagement would limit their exploration of the task dynamics, thereby decreasing their ability to learn these task dynamics. This suggests a nonlinear relationship between individual learning capabilities and the partner’s proficiency, where collaborating with a slightly better-performing partner may enhance learning, while a considerably better partner may hinder it. Additionally, pairs with the same handedness exhibited better collaboration performance. This contrasts with findings reported in previous research^43^, where it was concluded that the spatial configuration of participants (face-to-face vs. side-by-side) affected collaboration performance in a joint object manipulation task while handedness did not. Unlike this earlier study^43^, our experiment included both same- and different-handedness pairings while maintaining a face-to-face configuration. Therefore, it was possible to examine the influence of handedness differences on collaboration performance without varying other task conditions. In our study, all participants were required to use their right hand, meaning left-handed participants had to complete the task with their non-dominant hand. This might suggest that the observed effect could be due to participants performing worse with their non-dominant hand. However, our results indicate that participants’ handedness did not significantly affect solo task performance. An alternative possible interpretation is that performing the task with the non-dominant hand may require greater cognitive effort^44^, which could reduce this participant’s ability to effectively coordinate with the partner, thereby impairing overall collaboration performance in mixed-handedness pairs. This finding should be further investigated in future studies with larger and more balanced samples, potentially also incorporating objective measures of cognitive load to directly assess its role in mixed-handedness collaboration. These results emphasize the need to consider partner compatibility in collaborative settings, both to enhance performance and to maximize learning outcomes.

In summary, the study provides insights into the dynamics of human collaboration in high-precision tasks. Our findings suggest that collaboration performance improves as individuals gradually learn to improve the coordination of their actions through repeated interaction. This learning process may involve developing a predictive model of the partner’s contributions, enhancing predictability and coordination. Moreover, the specific collaboration partner influences both collaborative behavior and individual learning capabilities. Future studies should explore how these findings depend on task demands, such as precision requirements, range of motion, force demands, and amount of haptic feedback.

Our presented findings have the potential to enhance pHRC by designing robotic systems that aim to replicate human-like collaboration behavior. For instance, a robotic agent could gradually learn partner-specific interaction dynamics from repeated physical interactions with the same partner. By recalling these in future collaborations it may allow to achieve high performance immediately without requiring a retraining phase. Additionally, by leveraging the benefits of collaboration with a more skilled partner, robots could possibly be designed as expert teachers to enhance human performance in tasks requiring high precision, such as surgical training. Future research should explore what factors influence knowledge transfer during collaboration, including the impact of a wider range of performance differences. This could, for example, be tested in the collaborative hot wire task by conducting experiments with robotic agents of varying proficiency, such as different levels of trajectory tracking accuracy. These potential applications of our findings to pHRC should be validated in future studies.

## Supplementary Information

Supplementary Information.

## Data Availability

Data collected during this study is available on request from the corresponding author.
