# Supplementary material for: Partner familiarity enhances performance in a manual precision task
Source: Sci Rep. 2025 Jul 2;15:23381. doi: 10.1038/s41598-025-03341-9 (PMC12222539; doi:10.1038/s41598-025-03341-9)
Supplement: Supplementary file 1 — Supplementary Information. [file 41598_2025_3341_MOESM1_ESM.pdf]

Supplementary Information

This document contains supplementary information for the main article. The provided tables detail the statistical analysis results obtained from the Linear Mixed-Effects Models (LMEMs) in terms of estimated fixed effect coefficients β, the standard error of these coefficients SE, the t-statistic with the associated degree of freedom $t(df)$, and the p-values.

Table S1. The LMEM of Eq. (6) is used to examine how trial number and block condition influence the score. In the analysis, the collaboration conditions A1 (Partner 1), A2 (Partner 1 repeat), and B (Partner 2) are considered. The table presents the estimated fixed effect coefficients (β), their standard errors (SE), t-values, and p-values for each fixed effect.

**Fixed Effect**	β	SE	**t-value**	**p-value**
$\text{Condition}_{\text{A1},k}$	$\beta_{\text{A1}} = 50.29$	3.86	$t(982) = 13.01$	$p < 0.001$
$\text{Condition}_{\text{A2},k}$	$\beta_{\text{A2}} = 73.38$	3.87	$t(982) = 18.98$	$p < 0.001$
$\text{Condition}_{\text{B},k}$	$\beta_{\text{B}} = 60.49$	3.86	$t(982) = 15.65$	$p < 0.001$
$\text{Trial}_l\ \text{Condition}_{\text{A1},k}$	$\beta_{\text{A1,Trial}} = 0.97$	0.16	$t(982) = 5.92$	$p < 0.001$
$\text{Trial}_l\ \text{Condition}_{\text{A2},k}$	$\beta_{\text{A2,Trial}} = -0.06$	0.16	$t(982) = -0.39$	$p = 0.70$
$\text{Trial}_l\ \text{Condition}_{\text{B},k}$	$\beta_{\text{B,Trial}} = 0.76$	0.16	$t(982) = 4.66$	$p < 0.001$

Table S2. The LMEM with Eq. (7) is used to examine how consecutive motion dissimilarity changes over trial number. For each dissimilarity metric d_{s} a separate LMEM is used. The table lists the estimated fixed effect coefficients (β), their standard errors (SE), t-values, and p-values for each fixed effect.

**Response Variable**	β	SE	**t-value**	**p-value**
$d_{\text{s},k\hat{l}m}^{\text{Handle,Position}}$	$\beta_{\text{Trial}} = 0.02\,\text{mm}$	$2.55 \times 10^{-3}\,\text{mm}$	$t(888) = 9.30$	$p < 0.001$
$d_{\text{s},k\hat{l}m}^{\text{Hand,Position}}$	$\beta_{\text{Trial}} = -0.17\,\text{mm}$	$6.06 \times 10^{-2}\,\text{mm}$	$t(868) = -2.78$	$p = 0.006$
$d_{\text{s},k\hat{l}m}^{,\text{Wrist,Position}}$	$\beta_{\text{Trial}} = -0.30\,\text{mm}$	$8.88 \times 10^{-2}\,\text{mm}$	$t(831) = -3.42$	$p < 0.001$

Table S3. To examine how consecutive motion dissimilarity d_{s} influences the score, the LMEM from Eq. (6) is extended with an additional fixed effect for d_{s}. A separate LMEM is used for each dissimilarity metric d_{s}. The table lists the estimated fixed effects coefficients (β), their standard errors (SE), t-values, and p-values for the fixed effects.

**Fixed Effect**	β	SE	**t-value**	**p-value**
$d_{\text{s},k\hat{l}m}^{\text{Handle,Position}}$	$\beta_{\text{s}}^{\text{Handle,Position}} = -7.77\,\text{mm}^{-1}$	$1.23\,\text{mm}^{-1}$	$t(883) = -6.33$	$p < 0.001$
$d_{\text{s},k\hat{l}m}^{\text{Hand,Position}}$	$\beta_{\text{s}}^{\text{Hand,Position}} = -0.25\,\text{mm}^{-1}$	$0.05\,\text{mm}^{-1}$	$t(863) = -4.91$	$p < 0.001$
$d_{\text{s},k\hat{l}m}^{,\text{Wrist,Position}}$	$\beta_{\text{s}}^{\text{Wrist,Position}} = -0.11\,\text{mm}^{-1}$	$0.04\,\text{mm}^{-1}$	$t(826) = -2.86$	$p = 0.005$

Table S4. The LMEM with Eq. (8) is used to examine how similar times series from conditions A2 (Partner 1 repeat) and B (Partner 2) are to time series from the last trial of condition A1 (Partner 1). The table presents the estimated fixed effects coefficients (β), their standard errors (SE), t-values, and p-values for each fixed effect.

Response Variable	β	SE	t-value	p-value
$d^{\text{Force}}_{\text{c},\hat{k}lm}$	$\beta^{\text{Force}}_{\text{A2}} = 1.14\,\text{N}$	$0.14\,\text{N}$	$t(328) = 7.97$	$p < 0.001$
	$\beta^{\text{Force}}_{\text{B}} = 1.53\,\text{N}$	$0.14\,\text{N}$	$t(328) = 10.66$	$p < 0.001$
$d^{\text{Handle,Orientation}}_{\text{c},\hat{k}lm}$	$\beta^{\text{Handle,Orientation}}_{\text{A2}} = 11.43°$	$0.97°$	$t(321) = 11.75$	$p < 0.001$
	$\beta^{\text{Handle,Orientation}}_{\text{B}} = 17.25°$	$0.98°$	$t(321) = 17.66$	$p < 0.001$
$d^{\text{Handle,Position}}_{\text{c},\hat{k}lm}$	$\beta^{\text{Handle,Position}}_{\text{A2}} = 3.87\,\text{mm}$	$0.11\,\text{mm}$	$t(321) = 34.49$	$p < 0.001$
	$\beta^{\text{Handle,Position}}_{\text{B}} = 4.10\,\text{mm}$	$0.11\,\text{mm}$	$t(321) = 36.56$	$p < 0.001$
$d^{\text{Hand,Position}}_{\text{c},\hat{k}lm}$	$\beta^{\text{Hand,Position}}_{\text{A2}} = 32.18\,\text{mm}$	$2.74\,\text{mm}$	$t(554) = 11.73$	$p < 0.001$
	$\beta^{\text{Hand,Position}}_{\text{B}} = 34.44\,\text{mm}$	$2.75\,\text{mm}$	$t(554) = 12.53$	$p < 0.001$
$d^{\text{Wrist,Position}}_{\text{c},\hat{k}lm}$	$\beta^{\text{Wrist,Position}}_{\text{A2}} = 37.42\,\text{mm}$	$3.14\,\text{mm}$	$t(483) = 11.91$	$p < 0.001$
	$\beta^{\text{Wrist,Position}}_{\text{B}} = 38.46\,\text{mm}$	$3.14\,\text{mm}$	$t(483) = 12.23$	$p < 0.001$

Table S5. The LMEM in Eq. (10) is used to examine whether handedness affects solo task performance. The table lists the estimated fixed-effect coefficients (β) for each handedness type, their standard errors (SE), t-values, degrees of freedom, and p-values.

Fixed Effect	β	SE	t-value	p-value
$\text{Hand}_{\text{right},n}$	$\beta_{\text{right}} = 69.00$	6.00	$t(436) = 11.50$	$p < 0.001$
$\text{Hand}_{\text{left},n}$	$\beta_{\text{left}} = 61.11$	8.67	$t(436) = 7.05$	$p < 0.001$
$\text{Hand}_{\text{amb},n}$	$\beta_{\text{amb}} = 83.20$	19.16	$t(436) = 4.34$	$p < 0.001$
